# Emerging role of selective autophagy in human diseases

**DOI:** 10.3389/fphar.2014.00244

**Published:** 2014-11-05

**Authors:** Kenji Mizumura, Augustine M. K. Choi, Stefan W. Ryter

**Affiliations:** Joan and Sanford I. Weill Department of Medicine, Weill Cornell Medical Center, New York-Presbyterian Hospital – Weill Cornell Medical CollegeNew York, NY, USA

**Keywords:** autophagy, mitophagy, lung diseases, ciliophagy, xenophagy

## Abstract

Autophagy was originally described as a highly conserved system for the degradation of cytosol through a lysosome-dependent pathway. In response to starvation, autophagy degrades organelles and proteins to provide metabolites and energy for its pro-survival effects. Autophagy is recognized as playing a role in the pathogenesis of disease either directly or indirectly, through the regulation of vital processes such as programmed cell death, inflammation, and adaptive immune mechanisms. Recent studies have demonstrated that autophagy is not only a simple metabolite recycling system, but also has the ability to degrade specific cellular targets, such as mitochondria, cilia, and invading bacteria. In addition, selective autophagy has also been implicated in vesicle trafficking pathways, with potential roles in secretion and other intracellular transport processes. Selective autophagy has drawn the attention of researchers because of its potential importance in clinical diseases. Therapeutic strategies to target selective autophagy rather than general autophagy may maximize clinical benefit by enhancing selectivity. In this review, we outline the principle components of selective autophagy processes and their emerging importance in human disease, with an emphasis on pulmonary diseases.

## INTRODUCTION

Autophagy is a lysosomal degradation system by which the cell can recycle its cytoplasmic components (Mizushima and Komatsu, 2011). At present, three different types of autophagic pathways have been reported, named as macroautophagy, microautophagy, and chaperone-mediated autophagy (Mizushima and Komatsu, 2011). Of these, macroautophagy is the best-characterized and most well-known form, often referred to simply as “autophagy.”

Autophagy proceeds through sequential steps, beginning with the generation of autophagosomes from an isolation membrane and followed by elongation to form a mature autophagosome which captures cytosolic cargo (Mizushima and Komatsu, 2011). Genetic studies in yeast have identified a series of autophagy-related genes (ATGs) shown to be essential for the autophagy process (Tsukada and Ohsumi, 1993; Klionsky et al., 2003). Among these, microtubule associated protein 1 light chain-3 (LC3), a homologue of yeast Atg8, is converted from a cytosolic form (LC3-I) to its phosphatidylethanolamine-conjugated form (LC3-II) which targets to autophagic membranes (Mizushima et al., 2010). Autophagosome formation is also regulated by the autophagy protein Beclin 1 (homolog of yeast Atg6; Liang et al., 1999).

The membrane origin of autophagosomes remains unclear. Although the endoplasmic reticulum (ER), mitochondria and plasma membrane have been reported as the membrane source, recent studies have also suggested that the ER-mitochondria contact site is important in autophagosome formation (Hailey et al., 2010; Ravikumar et al., 2010; Tooze and Yoshimori, 2010; Hamasaki et al., 2013). Subsequently, the autophagosome containing the cytosolic components and organelles fuses with the lysosome to form the autolysosome where the sequestered cargo is degraded (Mizushima and Komatsu, 2011). Initial studies on the molecular mechanisms of autophagy have largely focused on the early stage, however, precise mechanisms of the late stage where the autophagosome fuses with the lysosome have also been revealed (Shen and Mizushima, 2014). Recent studies have demonstrated that the transcription factor EB (TFEB), a master gene for lysosomal biogenesis, coordinates the autophagic process by driving expression of autophagy and lysosomal related genes (Settembre et al., 2011). An autophagosomal soluble *N*-ethylmaleimide-sensitive factor attachment protein receptor (SNARE) has been identified as the regulator of autophagosome–lysosome fusion (Itakura et al., 2012).

Once, autophagy was simply regarded as a non-specific degradation system, however, recent research shows that autophagy can selectively degrade specific targets in processes referred to as “selective autophagy” (Levine et al., 2011; Youle and Narendra, 2011). Each selective autophagy subtype was named after its specific targets, for example: aggregated proteins (aggrephagy; Yamamoto and Simonsen, 2011), mitochondria (mitophagy; Youle and Narendra, 2011), pathogens (xenophagy; Levine et al., 2011), and cilia (ciliophagy; Cloonan et al., 2014). Selective autophagy is also related to vesicle trafficking pathways, and its importance in secretion and other intracellular transport processes is rapidly increasing (Stolz et al., 2014).

Previous studies suggest that autophagy is relevant to human diseases, including pulmonary diseases (Levine and Kroemer, 2008; Choi et al., 2013). Furthermore, convincing evidence that selective autophagy may be implicated in human disease has been reported (Gomes and Dikic, 2014; Redmann et al., 2014). This led us to the hypothesis that selective autophagy would impact the pathogenesis of pulmonary diseases. In this review, we will examine the considerable evidence emerging for the role of selective autophagy in the pathogenesis of complex pulmonary diseases. A better understanding of the role(s) of selective autophagy in disease pathogenesis may help design more specific therapies for the treatment of pulmonary diseases, and other diseases where autophagy may contribute to pathogenesis.

## SELECTIVE AUTOPHAGY

Selective autophagy can deliver a wide range of cargo to the lysosome, including protein aggregates, whole organelles (e.g., mitochondria), and intracellular pathogens (Stolz et al., 2014). Although the mechanisms of selective degradation remain incompletely understood, several reports suggest that ubiquitination of substrates may serve as general tag for selective autophagy in mammalian cells (Kirkin et al., 2009). Recent studies have described important functions of Atg8 family proteins in selective autophagy, including interactions with cargo receptors and components of the basal autophagy machinery, and in the regulation of autophagosome biogenesis (Kaufmann et al., 2014; Nath et al., 2014; Sawa-Makarska et al., 2014). To evaluate the inclusive list of selective autophagy processes currently in the literature is beyond the scope of this review; we will therefore focus on the three types of selective autophagy most related to pulmonary diseases; mitophagy, xenophagy, and ciliophagy.

### MITOPHAGY

Mitophagy is a selective mechanism for the elimination of mitochondria through the autophagic machinery (Youle and Narendra, 2011). Two major mitophagy-related proteins, Parkin and PTEN-induced putative kinase protein 1 (PINK1), have been linked to the pathogenesis of Parkinson’s disease (Youle and Narendra, 2011). A proposal for the mechanism of mitophagy is that damaged and depolarized mitochondria stabilize PINK1 which in turn recruits the E3 ubiquitin ligase, Parkin. Parkin then ubiquitinylates various mitochondrial outer membrane proteins including mitofusins MFN1, MFN2 (Gegg et al., 2010), voltage dependent anion channel (VDAC; Geisler et al., 2010) and mitochondrial rho GTPase (MIRO; Wang et al., 2011); and induces mitophagy by the recruitment of autophagy receptors such as p62 (Geisler et al., 2010). However, several previous reports are suggestive of PINK1-dependent, but Parkin-independent, mitophagy. Gp78 E3 ubiquitin ligase overexpression causes mitophagy that is independent of Parkin (Fu et al., 2013). Mice genetically deficient in Pink1 were resistant to *Staphylococcus aureus*-induced acute lung injury (ALI). PINK1 was found to interact with an alternative ubiquitin E3 ligase component, F-box only protein 15 (Fbxo15), which promoted mitochondrial instability in this model (Chen et al., 2014a). Furthermore, although mitophagy was generally considered to serve as an intrinsic mitochondrial quality control system, it has been reported that mitophagy may trigger cell death (Sentelle et al., 2012; Mizumura et al., 2014). Now, mitophagy is generally recognized as a potential modulator of the pathogenesis of disease with either protective or harmful consequences.

### XENOPHAGY

Autophagy can contribute to the immune response by providing a mechanism for the selective intracellular degradation of invading pathogens, a process termed “xenophagy.” Invading bacteria are tagged for removal with ubiquitin. Autophagy receptors including p62, nuclear domain 10 protein 52 (NDP52) and optineurin recognize ubiquitinated pathogens and target them to autophagosomes (Gomes and Dikic, 2014). Besides its direct role in pathogen clearance, xenophagy may also serve host defenses by enhancing immune recognition of infected cells via the generation of antigenic bacterial peptides (Yano and Kurata, 2011). Meanwhile, some bacteria, such as *S. aureus* and *Anaplasma phagocytophilum*, can use the host autophagosomes for replication (Schnaith et al., 2007; Niu et al., 2008). These bacteria can not only block autophagosomal maturation and acidification, but also can proliferate in LC3-positive compartments (Gomes and Dikic, 2014).

### CILIOPHAGY

Recently, we have demonstrated that an autophagy-dependent pathway regulates cilia length (Lam et al., 2013), a process named “ciliophagy.” We have shown that the cytosolic deacetylase histone deacetylase 6 (HDAC6) mediates ciliophagy. Pampliega et al. (2013) also reported that autophagy negatively regulate ciliogenesis by degrading intraflagellar transport protein 20 homolog (IFT20). On the other hand, it has reported that autophagy removes oral-facial-digital syndrome 1 protein (OFD1) from centriolar satellites to promote ciliogenesis (Tang et al., 2013). However, the precise mechanisms by which autophagy can regulate these conflicting processes remains to be elucidated (Wrighton, 2013).

## SELECTIVE AUTOPHAGY IN COPD

**FIGURE 1:**
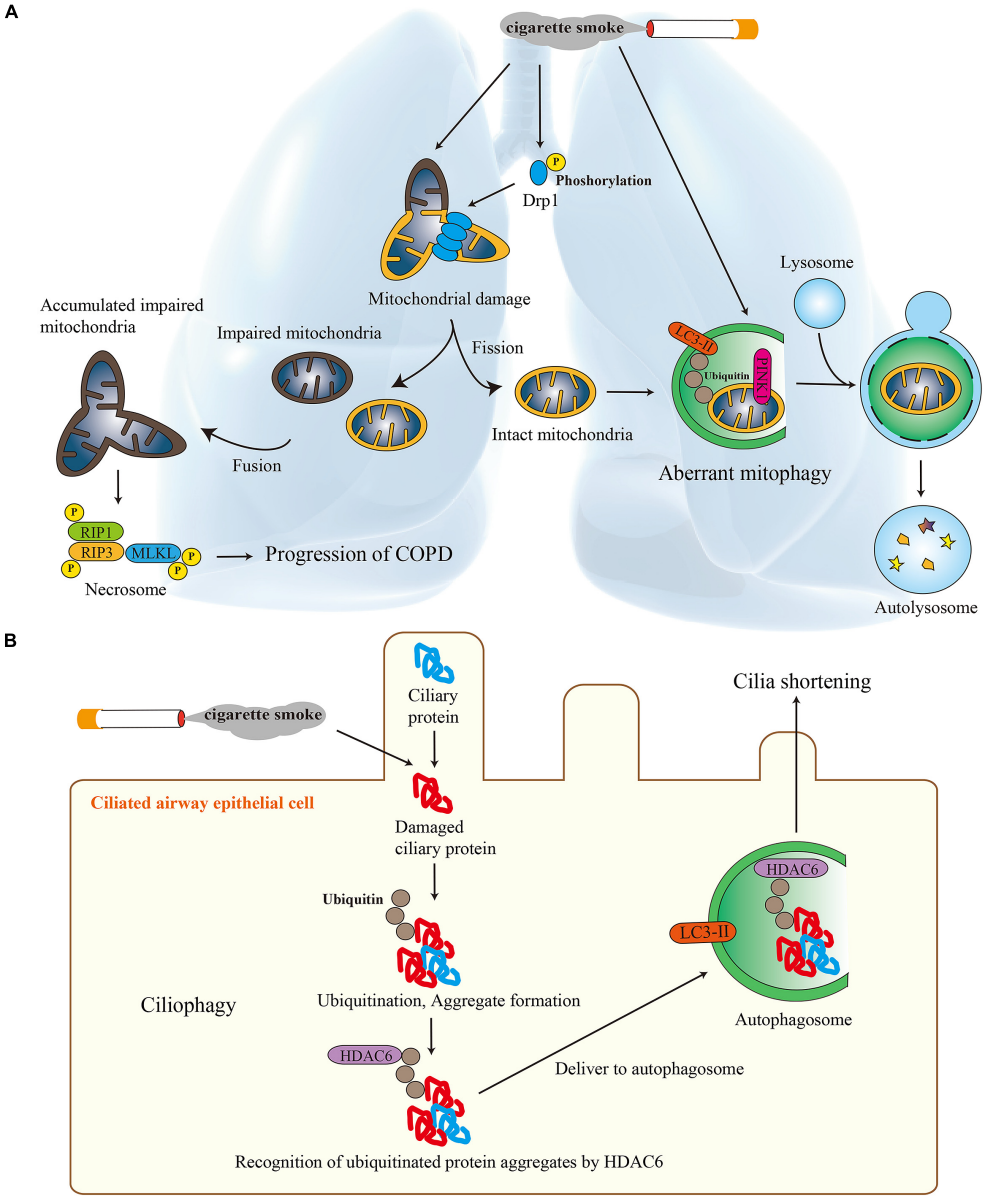
**Selective autophagy in chronic obstructive pulmonary disease (COPD). (A)** The role of mitophagy in COPD. Cigarette smoke (CS) induced mitochondrial fission and PINK1-dependent mitophagy in epithelial cells independently from mitochondrial damage. This aberrant mitophagy may cause the increase in the population of impaired mitochondria, which leads to the initiation of necroptosis. **(B)** The role of ciliophagy in COPD. CS induces oxidative stress, which causes cilia protein damage. Damaged cilia proteins are ubiquitinated which promotes aggregate formation. HDAC6 recognizes ubiquitinated protein aggregates and delivers them to autophagosomes. This degradation of cilia proteins, through an autophagy-dependent process termed “ciliophagy,” was associated with cilia shortening.

Chronic obstructive pulmonary disease (COPD) contributes significantly to the global burden of disease as the fourth leading cause of mortality worldwide, however, the pathogenesis of this disease remains incompletely understood (Dal-Re, 2011; Vestbo et al., 2013). We previously reported increased autophagosome numbers and increased expression of LC3B-II, the active form of LC3B, in human lung specimens from patients with COPD (Chen et al., 2010). In an *in vivo* emphysema model, genetic deletion of specific autophagy proteins reduced airspace enlargement (Chen et al., 2010). More recently, we demonstrated that mitophagy regulates necroptosis, a form of programmed necrosis, which contributes to the pathogenesis of COPD (Mizumura et al., 2014). Cigarette smoke (CS) exposure induced mitophagy through the stabilization of the mitophagy regulator PINK1 in pulmonary epithelial cells. Mice genetically deficient in PINK1 were protected against mitochondrial dysfunction, airspace enlargement, and mucociliary clearance (MCC) disruption during CS exposure. The mitochondrial division/mitophagy inhibitor Mdivi-1 protected against CS-induced cell death and mitochondrial dysfunction, and reduced the phosphorylation of mixed lineage kinase domain-like protein (MLKL), a substrate for receptor-interacting serine/threonine-protein kinase 3 (RIP3) in the necroptosis pathway. In this study, we have shown that significant mitochondrial depolarization occurred in pulmonary epithelial cells in response to CS extract (CSE) exposure. Moreover, our results suggest that active mitophagy may alter mitochondrial membrane integrity, and lead to the induction of necroptosis. However, the precise mechanism by which mitophagy can serve to aggravate mitochondrial injury in the CS exposure model remains obscure. One possible hypothesis is that CS-induced aberrant mitophagy may cause an increase in the population of impaired mitochondria (**Figure 1A**). In addition, as dose-response effects of autophagy/mitophagy have been proposed, we cannot completely exclude the possibility that mitophagy may also contribute to mitochondrial quality control for its pro-survival role during mild CS exposure (Frank et al., 2012; Sureshbabu and Bhandari, 2013; Schiavi and Ventura, 2014). Further studies are necessary to improve the understanding of the role of mitophagy in the pathogenesis of COPD.

We also reported that ciliophagy, the consumption of cilia components by autophagy, regulates cilia length during CS exposure (**Figure 1B**; Lam et al., 2013; Cloonan et al., 2014). Impaired airway clearance caused by cilia shortening prevents the elimination of pathogens from the airways and may cause recurrent respiratory infections that exacerbate COPD. We demonstrated that autophagy-impaired (*Becn1*^+/-^ or *Map1lc3B*^-/-^) mice, as well as tracheal epithelial cells isolated from these mice, resisted CS-induced cilia shortening. We identified the cytosolic deacetylase HDAC6 as a critical regulator of autophagy-mediated cilia shortening during CS exposure (Lam et al., 2013; Cloonan et al., 2014).

In contrast, previous studies have demonstrated defective autophagy in CS-exposed macrophages (Monick et al., 2010). Such a deficit in autophagy/xenophagy was observed in the alveolar macrophages of smokers and was proposed to lead to recurrent infections in smokers, since CS exposure impairs delivery of bacteria to the lysosomes.

## SELECTIVE AUTOPHAGY IN RESPIRATORY INFECTION AND SEPSIS

**FIGURE 2:**
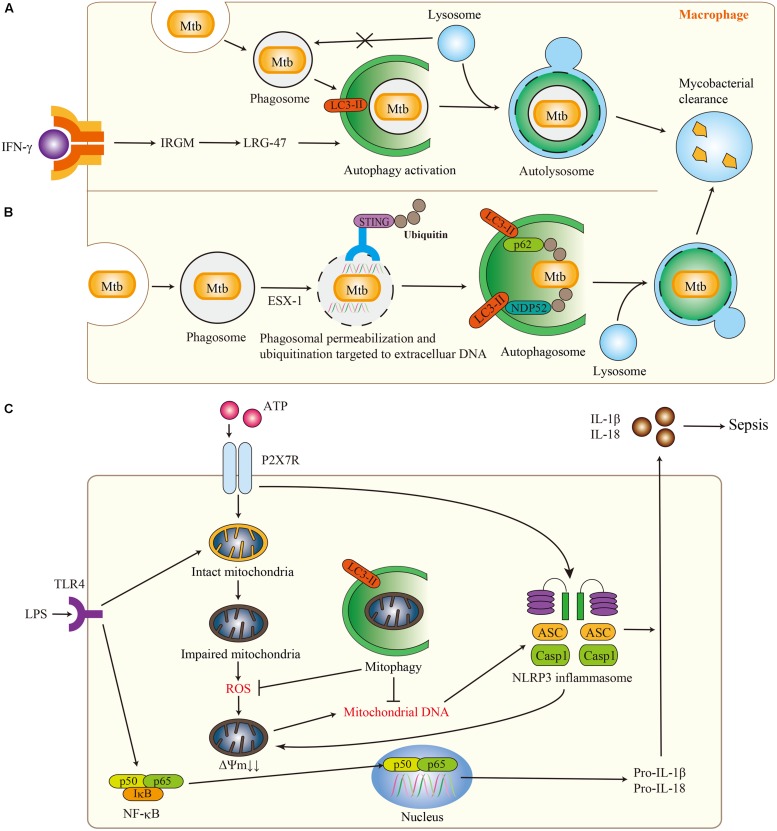
**Selective autophagy in respiratory infection and sepsis. (A)** The role of autophagy in *Mycobacterium tuberculosis* (Mtb) infection. IFN-γ induced IRGM activates autophagy and eliminates Mtb to outstrip the interference of fusion between phagosomal compartments containing Mtb and lysosomes. **(B)** The role of selective autophagy in Mtb infection. The bacterial ESX-1 secretion system mediates phagosomal permeabilization to enable the ubiquitin-mediated autophagy pathway to access phagosomal Mtb. Ubiquitinated Mtb is recognized by p62 and NDP52 as a target of selective autophagy. **(C)** The role of mitophagy in sepsis. The release of mitochondrial DNA (mtDNA) into the cytosol depends on the NLRP3 inflammasome and mitochondrial ROS. Cytosolic mtDNA contributes to the secretion of IL-1β and IL-18 in response to LPS and ATP. Mitophagy limits the secretion of IL-1β and IL-18 by targeting impaired mitochondria.

The mechanism of antibacterial autophagy in *Mycobacterium tuberculosis* (Mtb) infection is well-characterized. The lungs are the major site for Mtb infection. Mtb employs a unique strategy for survival that interferes with the fusion between phagosomal compartments containing Mtb and lysosomes (Vergne et al., 2004). Despite the availability of anti-TB drugs, recent reports have identified cases of totally drug-resistant TB (Loewenberg, 2012; Udwadia et al., 2012). Since new therapeutic agents that have different mechanisms of action from conventional anti-TB drugs are needed to prevent the development of drug resistance, bacterial autophagy (xenophagy) has drawn attention as a candidate therapeutic target. Previous studies have demonstrated that polymorphisms in the immunity-related GTPase family M protein (IRGM) gene are linked to increased susceptibility to Mtb infection, and that IFN-γ induced IRGM regulates autophagy to eliminate mycobacteria in human macrophages (**Figure 2A**; Singh et al., 2006; Intemann et al., 2009). Recent studies have revealed that Mtb extracellular DNA activates ubiquitin-mediated selective autophagy through phagosomal permeabilization (**Figure 2B**; Watson et al., 2012). The bacterial early secretory antigenic target 6 (ESAT-6) system 1 (ESX-1) secretion system mediates phagosomal permeabilization to enable the ubiquitin-mediated autophagy pathway access to phagosomal Mtb. The stimulator of interferon genes (STING)-dependent cytosolic pathway recognizes extracellular bacterial DNA and tags bacteria with ubiquitin. Autophagy receptors, p62, and NDP52, recognize ubiquitinated Mtb and target them to autophagosomes. Several therapies that involve enhancing autophagy activity also have been proposed to be effective against Mtb infection. The antiprotozoal drug nitazoxanide and its active metabolite tizoxanide strongly stimulate autophagy through inhibition of mTORC1 signaling, which in turn prevents intracellular proliferation of Mtb (Lam et al., 2012). Vitamin D has revealed therapeutic benefits in persons with HIV and Mtb infection through the activation of autophagy (Campbell and Spector, 2012).

Autophagy has been implicated in the regulation of inflammation, particularly the regulation of the inflammasome pathway. Inflammasomes represent an inflammatory signaling platform activated by infection or stress that regulate the maturation and secretion of pro-inflammatory cytokines (e.g., IL-1β and IL-18; Schroder and Tschopp, 2010). Along with Zhou et al, we have demonstrated that suppression of autophagy causes the accumulation of damaged ROS-producing mitochondria, whereas activates the NLRP3 inflammasome (**Figure 2C**; Nakahira et al., 2011; Zhou et al., 2011). In this study, we also found that the NLRP3 inflammasome and mitochondrial ROS production regulate cytosolic translocation of mitochondrial DNA (mtDNA) in macrophages, which in turn contributed to the secretion of IL-1β and IL-18. Importantly, these findings are consistent with the observation that IL-1β and IL-18 are increased in patients with sepsis in the medical intensive care unit (ICU; Nakahira et al., 2011; Dolinay et al., 2012). Moreover, we demonstrated that increased mtDNA levels in plasma are associated with ICU mortality, and inclusion of mtDNA level improves risk prediction in medical ICU patients (Nakahira et al., 2013). Recently, we have also demonstrated that carbon monoxide (CO) confers protection in sepsis by enhancing Beclin 1-dependent autophagy and phagocytosis (Lee et al., 2014). CO enhanced bacterial phagocytosis in *Becn1*^+/+^ but not *Becn1*^+/-^ mice *in vivo* and in corresponding cultured macrophages, which indicates that CO may induce xenophagy. These results suggest that CO gas may represent a novel therapy for patients with sepsis.

## SELECTIVE AUTOPHAGY IN HYPEROXIA AND ACUTE LUNG INJURY

We have demonstrated that autophagy is implicated in the pathogenesis of ALI (Tanaka et al., 2012). Although mechanical ventilation with high concentrations of oxygen is required to manage patients with severe respiratory failure, prolonged exposure to hyperoxia can result in lung injury. Hyperoxia can induce autophagy activity. Depletion of LC3B by RNA interference reduced cell viability under hyperoxic conditions (Tanaka et al., 2012). We also investigated the molecular mechanism by which autophagy can confer cytoprotection in lung epithelial cells after hyperoxia (Liang et al., 2013). Cellular homeostasis requires the constant formation of the p62/LC3B/truncated BH3-interacting domain death agonist (tBID) complex under normal conditions, however, hyperoxia leads to dissociation of the p62/LC3B/tBID complex, which stops the translocation of tBID into lysosome for degradation. Increased tBID causes cytochrome c release from the mitochondria and subsequent caspase-dependent cell death. These results suggest that the autophagy may have protective function during the pathogenesis of ALI, especially under hyperoxia.

It has been reported that mechanical ventilation and hyperoxia cause pulmonary mitochondrial dysfunction (Ratner et al., 2009, 2013; Waxman and Kolliputi, 2009). Given that mitochondrial damage can induce mitophagy, it is reasonable to presume that mitophagy may play a role in the pathogenesis of ALI (Youle and Narendra, 2011). Indeed, the role of mitophagy in hyperoxia has been reported. Genetic deletion of PINK1 or PINK1 silencing in the lung endothelium increased susceptibility to hyperoxia via alterations in autophagy/mitophagy (Zhang et al., 2014). NLRP3 may regulate autophagy/mitophagy via PINK1 during hyperoxia. Consistent with a role for autophagy, these results also suggest that mitophagy may have protective function during the pathogenesis of ALI.

## SELECTIVE AUTOPHAGY IN THE HYPOXIA RESPONSE AND PULMONARY HYPERTENSION

Hypoxia results in secondary pulmonary hypertension (PH). Hypoxic PH is a progressive and often fatal complication of chronic lung disease (Semenza, 2011). Chronic hypoxia induces pulmonary arterial vascular smooth muscle (PAVSM) cell proliferation, which is a major cause of PH (Stenmark et al., 2006). We previously demonstrated that elevated occurrences of autophagy have been observed in lung tissue from patients with PH. Mice genetically deficient in LC3B demonstrated increased indices of PH after chronic hypoxia (Lee et al., 2011). Chloroquine, the inhibitor of autophagy, has been reported to prevent progression of experimental PH (Long et al., 2013). Furthermore, as the inhibition of mTOR complex 1 (mTORC1) has been shown to induce autophagy, previous studies demonstrated that blockade of mTORC1 has anti-proliferative effects on pulmonary vascular cells (Krymskaya et al., 2011; Wang et al., 2014). These results suggest that autophagy may have a protective function during the pathogenesis of PH. LC3 and mTOR pathway are attracting attention as potential therapeutic targets in hypoxia-induced PH (Lahm and Petrache, 2012; Goncharova, 2013).

The mitochondrial outer-membrane protein FUN14 domain-containing protein 1 (FUNDC1) mediates hypoxia-induced mitophagy in mammalian cells (Liu et al., 2012). FUNDC1 interacted with LC3; and knockdown of endogenous FUNDC1 significantly prevented hypoxia-induced mitophagy, which could be reversed by the expression of wild-type FUNDC1. Hypoxia can dephosphorylate FUNDC1 at serine 13 through serine/threonine-protein phosphatase PGAM5 for the induction of mitophagy (Chen et al., 2014b; Wu et al., 2014). These results suggest that mitophagy may contribute to mitochondrial quality control in hypoxia. However, hypoxia-induced mitophagy has also been reported to cause apoptosis in cardiomyocytes (Yan et al., 2014). The role of mitophagy in the pathogenesis of PH remained incompletely understood.

## CONCLUSION

Although autophagy originally was considered as a simple bulk degradation system for cellular components, accumulating evidence demonstrates that autophagy can selectively degrade specific targets. Selective autophagy plays a complex role in human diseases where it can have both protective and injurious effects. However, when viewed in the light of evidence that many cellular functions can have both protective and injurious effects, it is likely that selective autophagy may also act as a double-edged-sword in the pathogenesis of human diseases. For future clinical applications, rather than intervention strategies to target general autophagy, the specific targeting of selective autophagy pathways may enhance the efficacy of therapeutic strategies. Further research into selective autophagy in the lung and other organs will allow for the development new therapeutic interventions.

## Conflict of Interest Statement

The authors declare that the research was conducted in the absence of any commercial or financial relationships that could be construed as a potential conflict of interest.
